# Reviewer List

**DOI:** 10.4269/ajtmh.94-01rev

**Published:** 2016-01-06

The journal would like to recognize the following individuals who have reviewed manuscripts during the past year. We thank you for your continued support.

Aaskov, John

Abdeladhim, Maha

Abeles, Shira

Abrahao, Jonatas

Aceituno, Anna

Achee, Nichole

Adamson, Martin. A

Adhikarla, Haritha

Agampodi, Suneth

Agudelo-Flórez, Piedad

Aguilar, Patricia

Ahmed, Ahmed

Aidoo, Michael

Aiello, Allison

Ajjampur, Sitara

Akanda, Ali

Al-Mekhlafi, Hesham

Alborzi, Abdolvahab

Albrecht, Randy

Alcid, David

Aldridge, Jerry

Aldstadt, Jared

Alegana, Victor

Alevi, Kaio Cesar

Ali, Mohammad

Almeida, Angela Freitas C.

Almeida, Carlos

Almeida, Roque

Althaus, Thomas

Alves, Giordano

Amaral, Veronica

Anders, Katherine

Anderson, John

Anderson, Kathryn

Andrade, Christy

Andrews, Jason

Apperson, Charles

Araujo, Wildo

Arce-Fonseca, Minerva

Arguin, Paul

Arinaitwe, Emmanuel

Armbruster, Peter

Armien, Blas

Armstrong, Philip

Arnold, Benjamin

Aronson, Naomi

Arrigo, Nicole

Asahi, Hiroko

Ash, Lawrence

Asiedu, Kingsley

Ataide, Ricardo

Auer, Herbert

Aung, Ar Kar

Aviles, Maria

Aweeka, Fran

Axon, Anthony

Aydin-Schmidt, Berit

Babiker, Hamza

Bacon, David

Bain, Rob

Baine, William

Bancone, Germana

Bao, Changiun

Barat, Lawrence

Barbour, Alan

Barlow, Jon

Barral-Netto, Manoel

Barrera, Roberto

Barrett, Alan

Barros, Elvino

Bart, Jean-Mathieu

Basile, Jane

Basombrío, Miguel

Bassat, Quique

Bastos, Claudilson

Bates, Paul A.

Battle, Katherine

Baugh, Gunther

Baumann, Francine

Bausch, Daniel

Bausch, Daniel

Bautista, Christian

Beasley, David

Beau de Rochars, Valery

Beissner, Marcus

Bejon, Philip

Bekal, Sadjia

Benard, Gil

Benbow, Eric

Benedict, Mark

Benjamin-Chung, Jade

Bennett, Adam

Bennett, Malcolm

Berens-Riha, Nicole

Berg, Douglas

Berman, Jonathan

Bern, Caryn

Beverley, Steve

Beyer, Wolfgang

Bhattarai, Rachana

Bird, Brian

Biritwum, Nana Kwadwo

Bitam, Idir

Björnsson, Einar S.

Black, William

Blacksell, Stuart

Blair, Carol

Blaney, David

Blaney, David

Blanton, Lucas

Bleeker-Rovers, Chantal

Blitvich, Bradley

Blosser, Erik

Boakye, Daniel

Boateng, Joshua

Bocanegra, Cristina

Bodeau-Livinec, Florence

Boehme, Catharina

Bogoch, Isaac

Bogus, Joshua

Boite, Mariana

Bolling, Bethany

Boom, W.

Bottasso, Oscar

Bottieau, Emmanuel

Boulware, David

Bousema, Teun

Boussinesq, Michel

Bowen, Asha

Bowen, Richard

Bowman, Dwight

Bradley, Jan

Bradley, Kristy

Brady, Oliver

Brandão, Paulo

Brandling-Bennett, A.

Braslins, Philip

Brault, Aaron

Bravo, Francisco

Brazil, Reginaldo

Breman, Joel

Brennan, Patrick

Brett-Major, David

Bridson, Tahnee

Brieger, William

Brinckman, Robert

Britto, Constança

Brodskyn, Claudia

Brook, Itzhak

Brown, Katherine Anne

Brunetti, Enrico

Bruxvoort, Katia

Bryceson, Anthony

Brynjarsdóttir, Jenny

Buchanan, Ann

Buffet, Pierre

Burdmann, Emmanuel

Burza, Sakib

Bustamante, Dulce

Bustinduy, Amaya

Byass, Peter

Cabié, André

Cai, Li

caillouet, Kevin

Caldeira, Daniela

Calisher, Charles

Cama, Vitaliano

Campbell, Carl

Campbell, Carlos

Campbell, Corey

Campos-Neto, Antonio

Cannella, Anthony

Cao-Lormeau, Van-Mai

Cao, Chang

Cao, Jun

Cao, Shengbo

Capobianchi, Maria Rosaria

Cardoso, Luis

Carmolli, Marya

Carneiro, Cláudia

Carrero Sánchez, Julio

Carrington, Christine

Carter, Keith

Carvalho Siqueira, Gabriela

Carvalho, Edgar

Casanova, Lisa

Castro, Vagner

Cattamanchi, Adithya

Caumes, Eric

Cavalcante, Ricardo S.

Cegielski, Peter

Cerritos, Rene

Chakrabarti, Sumontra

Chamie, Gabriel

Chang, KP

Charrel, Remi

Chaudhuri, Gautam

Checkley, Wlliam

Chen, Min-Hui

Chen, Rubing

Chen, Xinhua

Chen, Ya-Lei

Chieffi, Pedro

Ching, Wei-Mei

Chitsulo, Lester

Cho, Nam-Hyuk

Choi, Mary

Christinet, Vanessa

Christofferson, Rebecca

Chunara, Rumi

Chuwuma, Adanna

Clarke, Sian

Clennon, Julie

Coetzee, Maureen

Coffey, Lark

Cohen, Craig

Cohen, Justin

Colborn, James

Colgate, E.

Colombo, Mauro

Conn, Bruce

Conn, Jan

Connor, Patrick

Conroy, Ronán

Cooper, Roland

Cope, Jennifer

Courtenay, Orin

Courtin, David

Coyle, Christine

Coyne, Philip

Craig, Alister

Cramer, Jakob P.

Crozier, Ian

Cui, Liwang

Cunha-Neto, Edecio

Cupolillo, Elisa

Currie, Bart

Curtis, Val

Cutler, Sally

da Silva-Nunes, Mônica

Dahlgren, Fredrick

Daily, Johanna

Dance, David

Daniels, Rachel

Dann, Sara

Darbro, Jon

Darius, Taiana

Das, Murari

Das, Pradeep

Dasch, Gregory

David, John

Day, Nick

DeCamp, Matthew

Dedicoat, Martin

Del Brutto, Oscar

della Torre, Alessandra

Delmondes, Leda

Deribe, Kebede

Devine, Greg

Diez, Cristina

Dittrich, Sabine

Djimde, Abdoulaye

Dobson, Stephen

Doganay, Mehmet

Dolmans, Wil

Domingo, Gonzalo

Doolan, Denise

Dorsey, Grant

Douglass, Richard

Downs, Jennifer

Drakeley, Chris

Drebot, Mike

Duffy, Craig

Duggal, Nisha

Duggal, Priya

Dumler, J Stephen

Dupont, Herbert

Durvasula, Ravi

Duthie, Malcolm

Dyrhol Riise, Anne Margarita

E. Lezi

Ebel, Gregory

Eberhard, Mark

Ebihara, Hideki

Edwardson, Jill

Eisele, Thomas

El-Sayed, Najib

Elder, John

Elinav, Hila

Elizabeth Joekes, Elizabeth

Ennis, Francis

Ephraim, Richard

Ephros, Moshe

Epstein, Judy

Escalante, Ananias

Evans, Darin

Ezeamama, Amara

Facchinelli, Luca

Faldetta, Kimberly

Felix, Todd

Fenwick, Alan

Fernández-Caldas, Enrique

Fernandez, Stefan

Fierer, Joshua

Firth, Jacqueline

Fischer, Marc

Fischer, Peter

Fischer, Philip

Fitchett, Joseph

Flannery, Luciana

Fletcher, Tom

Flisser, Ana

Fong, Mun

Fong, Raymond

Forrester, Naomi

Forshey, Brett

Foy, Brian

Fraga, Jorge

Freedman, David

Freeman, Matthew

Freitas, Simone

French, Neil

Friberg, Heather

Friedman, Jennifer

Fuentes, Isabel

Fuentes, Marius

Fuller, James

Fung, Isaac Chun-Hai

Gabriël, Sarah

Gaff, Holly

Gage, Kenneth

Galvan, Jose Manuel

Galvao, Lucia

Gamage, Chandika

Ganz, Holly

Gao, Lu

Garba, Amadou

García Bustos, María

Garcia, Hector

Garcia, Melissa

Garn, Joshua

Gass, Katie

Gatton, Michelle

GAUZERE, Bernard

Gee, Jay

Geissler, Paul Wenzel

Gelderblom, Huub

George, Christine

Gérardin, Patrick

Getis, Arthur

Ghumra, Ashfaq

Glass, Gregory

Gnanadurai, Clement

Godsey, Marvin

Goenka, Naila

Goethert, Heidi

Goldfarb, David

Goletti, Delia

Gonçalves, Bronner

Good, Michael

Goris, Marga

Gosling, Roly

Goswami, Doli

Goto, Hiro

Gottdenker, Nicole

Gotuzzo, Eduardo

Goulielmos, George

Grad, Yonatan

Gradoni, Luigi

Graff, Joel

Graham, Jay

Graves, Stephen

Graybill, John R.

Green, Michael

Green, Sharone

Gregory, Christopher

Gregson, Aric

Grogl, Max

Gromowski, Gregory

Gryseels, Charlotte

Guerin, Philippe

Guerra, Humberto

Guerra, Jorge

Guerra, Marta

Guerrant , Richard

Guhl, Felipe

Gute, David

Gutman, Julie

Guzman-Herrador, Bernardo Rafael

Gwack, Jin

Haddow, Andrew

Hall-Mendelin, Sonja

Hall, Andrew

Hall, Robert

Hall, Roy

Halsey, Eric

Halstead, Scott

Hamer, Davidson

Hanley, Kathryn

Harhay, Michael

Harley, David

Harris, Angela

Harris, Julie

Hatz, Christoph

Heitzinger, Kristen

Hemme, Ryan

Henao-Martínez, Andrés

Hermann, Emmanuel

Hickey, Patrick

Hifumi, Toru

Hildreth, Michael

Hill, Carlotta

Hill, Philip

Ho, Mitchell

Hodel, Eva Maria

Hoffman, Risa

Hoffman, Stephen

Hoffmaster, Alex

Hoft, Daniel

Hoglund, Richard

Holland, Celia

Holmen, Sigve

Hontz, Robert

Horstick, Olaf

Horton, John

Hospenthal, Duane

Houze, Sandrine

Hsiang, Michelle

Huang, Huan

Hubalek, Zdenek

Humphries, Debbie

Hunsperger, Elizabeth

Huston, Christopher

Hwang, Jimee

Idro, Richard

Ikegami, Tetsuro

Imanashi, Maho

Inglis, Timothy

Intapan, Pewpan

Ismail, Ahmad

Ito, Akira

Jackson, Alan

Jacobsen, Kathryn

Jaffe, Charles

Jansen, Ana

Javelle, Emilie

Jeronimo, Selma

Johansson, Anders

John, Chandy

Johnson, Barbara

Johnson, Heather

Johnson, Paul

Johnston, Rick

Jones, Andrew

Jones, James

Jutla, Antarpreet

Kahn, James

Kain, Kevin

Kaiser, Christoph

Kalayanarooj, Siripen

Kamar, Nassim

Kamau, Edwin

Kapur, Anil

Karim, Ali

Kaur, Harparkash

Kavanaugh, Michael

Kaye, Paul

Kayser, Georgia

Kazura, James

Keenan, Jeremy

Keep, Lisa

Kelly, Daryl

Kelly, Rosmarie

Kemperman, Melissa

Kenney, Joan

Kersh, Gil

Kersh, Gilbert

Keysary, Avi

Khalifeh, Ibrahim

Khan, Naveed Ahmed

Kim, Dohyeong

Kim, Dong-Min

Kima, Peter

Kimball, Ann Marie

King, Charles

Kirchhoff, Louis

Kirkland, Theo

Kirkpatrick, Beth

Kishimoto, Miori

Kjetland, Eyrun

Klein, Eli

Kleinschmidt, Immo

Klimstra, William

Klisiowicz, Debora

Kobayashi, Seiki

Kochel, Tadeusz

Koehler, Jane

Koenraadt, Sander

Koh, Gavin

Kolars, Joseph

Komar, Nicholas

Kortekaas, Jeroen

Kositchaiwat, Chomsri

Kosoy, Michael

Kotlyar, Simon

Koudou, Benjamin

Kozarsky, Phyllis

Kraemer, Moritz

Krammer, Florian

Krolewiecki, Alejandro

Kuhn, Walter

Kumagai, Takashi

Kung'u, Jacqueline

Kurt, Özgür

Kurup, Asok

Kutalek, Ruth

LaBeaud, Angelle

LaBeaud, Angelle Desiree

Lam, Tommy

Lambert, Amy

Lambert, Veronique

Lambrechts, Louis

Lammie, Patrick

Lanata, Claudio

Lankau, Emily

Lantagne, Daniele

Larson, Heidi

Lau, Audrey

Lau, Colleen

Laurenti, Marcia

Lecuit, Marc

Lee, Ben

Lee, Kim Sung

Legua, Pedro

Lehmann, Tovi

Leisnham, Paul

Lemey, Phiippe

Lengeler, Christian

Lenhart, Audrey

Leontsini, Elli

Leparc-Goffart, Isabelle

Lescano, Andres

Lessler, Justin

Levecke, Bruno

Levett, Paul

Levy, Karen

Lewis, Michael

Li, Jing

Liabsuetrakul, Tippawan

Libraty, Daniel

Licina, Derek

Lieberman, Marya

Lier, Tore

Lightowlers, Marshall

Lilje, Jonathan

Lim, Stephanie

Lima, Aldo

Limmathurotsakul, Direk

Lindo, John

Linhares, Alexandre

Linton, Yvonne

Liu, Jien-Wei

Llanos-Cuentas, Alejandro

Llinas, Manuel

Long, Kanya

López-Vélez, Rogelio

Lopman, Benjamin

Lortholary, Olivier

Lover, Andrew

LoVerde, Philip

Lubell, Yoel

Luby, Stephen

Lucas, Patricia

Luna, Expedito José

Lye, David

Lynch, Caroline

Lynch, Matthew

Lyra, Marcelo

Mabey, David

Machado, Paulo

Mackenzie, Charles

Madsen, Henry

Mahanty, Siddhartha

Manabe, Yukari

Maravilla, Pablo

Marcos, Luis

Maretti-Mira, Ana Claudia

Mariconti, Mara

Markoff, Lewis

Marks, Michael

Marsh, Kevin

Martin, Diana

Martínez, Homero

Marty, Francisco

Mathanga, Don

Matlashewski, Greg

Matsunaga, Jim

Matthias, Michael

Mattioli, Mia Catharine

Maves, Ryan

Mboowa, Gerald

Mbuagbaw, Lawrence

McAllister, Janet

McAvin, James

McCord, Gordon

McCormick, Ben

McElroy, Peter

McLean, Robert

McMahon-Pratt, Diane

McManus, Donald

Medlicott, Kate

Mehta, Sanjay

Melby, Peter

Meshnick, Steve

Messer, Bill

Meyer, Hermann

Miller, Nathan

Milord, Marie

Mintz, Eric

Mitjà, Oriol

Miura, Tanya

Molina, Israel

Molina, Ricardo

Monge-Maillo, Begoña

Monroy, Maria

Montgomery, Susan

Moonen, Bruno

Mooney, Sarah

Moore, Melinda

Moore, Thomas

Moraes Figueredo, Luiz Tadeu

Moraes, Milton

Morakote, Nimit

Morales-Hojas, Ramiro

Moran, Allisyn

Morand, Serge

Mori, Amani Thomas

Moro, Pedro

Morpeth, Susan

Morris, Aaron

Morris, Ulrika

Morrison, Amy

Morzunov, Sergey

Moss, William

Mourya, Devendra

Moyes, Catherine

Muljono, David

Murdoch, David

Murphy, Sean

Murray, Henry

Murray, Kristy

Murray, Melanie

Mwinzi, Pauline

Myler, Peter

Nacher, Mathieu

Nadjm, Behzad

Nahlen, Bernard

Nakazawa, Yoshinori

Namangala, Boniface

Namuyinga, Ruth

Nasci, Roger S.

Nash, Theodore

Nelson, Kenrad

Nery, José Augusto da Costa

Nevin, Remington

Newman, Peter

Newton, Paul

Niedrig, Matthias

Nieto, Nathan

Niyogi, Swapan Kumar

Njenga, Kariuki

Nolan, Thomas

Noordin, Rahmah

Norris, Douglas

Novaes Ramos, Alberto

Nutman, Thomas

O'Ryan, Miguel

Obare, Francis

Obaro, Stephen

Oberhelman, Richard

Ochoa, Maria

Odermatt, Peter

Ogden, Nicholas

Okeke, Iruka

Oliveira, Jader

Olkowski, Sandy

Olson, Ken

Ooi, Eng Eong

Ooi, Winnie

Orgill, Jenny

Osborn, Kent

Osier, Faith

Ostfeld, Richard

Ouellette, Marc

Outterson, Kevin

Owusu-Agyei, Seth

Page, Kathleen

Pallecchi, Lucia

Pang, Junxiong

Parajuli, Rajendra

Parikh, Sunil

Paris, Daniel

Park, Sang Won

Parry, Christopher

Patrick, Molly

Patterson, Jean

Paupy, Christophe

Pauvolid, Alex

Pavlinac, Patricia

Peeters Grietens, Koen

Peleteiro, Barbara

Peletz, Rachel

Pérez-Guerra, Carmen

Perez-Molina, Jose

Perez-Perez, Guillermo

Perng, Guey Chuen

Perry, Henry

Peter, Olivier

Petersen, Christine

Petersen, Lyle

Peterson, Stefan

Petri, William

Petruccelli, Bruno

Phanouvong, Souly

Pickering, Amy

Pion, Sébastien

Pisani, Elizabeth

Platts-Mills, James

Porco, Travis

Portell, Craig

Porter, Chad

Powers, Ann

Price, Erin

Price, Ric

Pruss-Ustun, Annette

Queiroz-Telles, Flavio

Quinnell, Rupert

Quiñones, Martha

Quinto, Llorenc

Rabaa, Maia

Rabe, Ingrid

Rabinovich, Regina

Radke, Elizabeth

Raffray, Loic

Rajasekariah, G-Halli

Ram, Pavani

Ramachandra-Rao, Satish

Ramachandran, Ranjini

Ramirez, Juan David

Rand, Emily

Ranson, Hilary

Raoult, Didier

Reddy, Elizabeth

Rego, Ryan

Reid, Gregor

Reid, Sean D.

Reimer, Lisa

Reiner, Robert

Reinharz, Daniel

Reisen, William K.

Reithinger, Richard

Reller, Megan

Renia, Laurent

Riano, Isolina

Ribeiro, Aline

Ribeiro, Guilherme

Ribeiro, Jose

Richards, Jack

Richie, Thomas

Riddle, Mark

Riley, Eleanor

Ritchie, Scott

Robertson, Gemma

Rodriguez-Barraquer, Isabel

Rodriguez-Morales, Alfonso

Rodriguez-Perez, Mario

Rodriguez, Isabel

Rodwell, Timothy

Roepe, Paul

Roess, Amira

Roestenberg, Meta

Rogerson, Stephen

Rolim, Dionne

Romero, Gustavo

Rosa, Ghislaine

Rosenberg, Ronald

Rosenthal, Philip

Roy, Syamal

Rubach, Matthew

Ruiz, Joaquim

Ruktanonchai, Nick

Ruperez, Maria

Russell, Tanya

Rutebemberwa, Elizeus

Ruybal, Paula

Ryan, Edward T.

Ryan, Peter

Sack, David

Sadruddin, Salim

Safronetz, David

Saha, Samir

Sakurai, Tatsuya

Salinas, Jorge

Salmon, Gabriela

Sampaio, Raimunda

Samy, Abdallah

Sandoval, Cesar

Sangenis, Luiz

Santos-Oliveira, Joanna

Sasaki, Mizuki

Saunders, David

Savage, Harry

Scharff, Robert

Schildgen, Oliver

Schiøler, Karin

Schlagenhauf, Patricia

Schmidt, Wolf-Peter

Schoeps, Anja

Schriefer, Albert

Schuh, Amy

Schultsz, Constance

Schurer, Janna

Schwan, Tom

Schwartzman, Joseph

Scollard, David

Scopel, Kezia

Scott, Alan

Scott, Thomas

Seas, Carlos

Sebastian, Miguel San

Secor, W. Evan

Secor, William

Seguro, Antonio

Sejvar, James

Sekiyama, Makiko

Sermswan, Rasana

Serre, David

Seshadri, Pratibha

Severson, David

Sevilimedu, Varadan

Shadomy, Sean

Shaheed, Ameer

Shanks, Dennis

Shanks, G.

Sharlow, Elizabeth

Sharp, Tyler

Shaw, Jeffrey

Shepard, Donald

Shih, James

Shivakoti, Rupak

Siedner, Mark

Simmerman, Mark

Simon, Gregory

Singer, Burton

Singh, Neeloo

Singh, SP

Siqueira, Andre

Sironen, Tarja

Sitprija, Visith

Slutsker, Laurence

Smart, Hiske

Smith, Darci

Smith, David

Smith, David

Smith, Tom

Smits, Henk

Smout, Michael

Snounou, Georges

Sokol, Lubomir

Song, Lijiang

Sorvillo, Frank

Sosa-Estani, Sergio

Sotillo, Javier

Southern, Paul

Sreeramareddy, Chandrashekhar

Srikiatkhachorn, Anon

St. Jeor, Stephen

Staedke, Sarah

Stamm, Lola

Stanisic, Danielle

Staples, J.

Steffen, Robert

Storberg, Viktor

Streicher, Elizabeth

Stromdahl, Ellen

Stuen, Snorre

Sullivan, David

Sun, Xi-Meng

Sutherland, Colin

Suzuki, Yasuhiro

T, Mahalakshmy

Tacchini-Cottier, Fabienne

Takken, William

Talhari, Sinesio

Tanowitz, Herbert

Taraphdar, Debjani

Tate, Jacqueline

Taylor, Diane

Taylor, Steve

Taylor, Terrie

Taylor, Walter

Teklehaimanot, Hailay

Tesh, Robert

Tetsuya, Iida

Thangamani, Saravanan

Thomas, Matthew

Thomas, Wayne

Tisch, Daniel

Toma, Helena

Tomczyk, Sara

Torgerson, Paul

Travi, Bruno

Trenholme, Katharine

Treurnicht, Florette

Triana Chavez, Omar

Tripet, Frederic

Troyo, Adriana

Trueba, Gabriel

Trung, Dinh

Tseng, Chien-tek

Tsetsarkin, Konstantin

Turell, Michael

Turnbull, Peter

Turner, Paul

Tyler, Kenneth

Tymchuk, Chris

Umemura, Takeji

Umezaki, Masahiro

Undurraga, Eduardo

Unnasch, Thomas

Utzinger, Juerg

Vaidya, Akhil

Valete-Rosalino, Claudia

Vallabheni, Snigdha

Vallejo, Gustavo

Van der Auwera, Gert

Van Rie, Annelies

van Steenbergen, Jim

Vasilakis, Nikolaos

Vaughan, Stephen

Vaughn, David

Vazquez, Gonzalo

Vekemans, Johan

Velavan, Dr. Thirumalaisamy

Verdonck, Kristien

Vignier, Nicolas

Villacís, Anita

Vinetz, Joseph

von Seidlein, Lorenz

Vuagnat, Hubert

Waggoner, Jesse

Walker, David

Walker, Ned

Walker, Patricia

Walker, Timothy

Walsh, Douglas

Walter, Katharine

Wanachiwanawin, Darawan

Wanji, Samuel

Warburg, Alon

Warrell, David

Wassmer, Samuel

Waterman, Stephen

Waters, Norman

Watt, George

Watts, Douglas

Weaver, Scott

Weil, Gary

Weina, Peter

Wendland, Claire

Wesche, David

Wheat, Joseph

White, A. Clinton

White, Michael

Whitehorn, Jamie

Whittaker, Maxine

Wilcox, Bruce

Wilder-Smith, Annelies

Williams, David

Willoughby, Rodney

Wilson, Anne

Wilson, Mary

Wimberly, Michael

Winkler, Cheryl A.

wiwanitkit, viroj

Won, Kimberly

Woodrow, Charlie

Wormser, Gary

Wright, James

Wright, Peter

Wu, Qingzhong

Wunner, W.

Wyles, David

Yakob, Laith

Yan, Guiyun

Yang, C.H.

Yasuda, Maria Aparecida

Yin, Shengyong

Yoon, In Kyu

Yoon, In-Kyu

Young, Ed

Young, Sara

Yu, Xue-Jie

Yuill, Thomas

Zammarchi, Lorenzo

Zhang, Ying

Zhu, Hongying

Zielinski-Gutierrez, Emily

Zimic, Mirko

Zimmerman, Peter

Zinsstag, Jacob

Zouache, Karima

Zulantay, Ines

Zúniga, Concepción

